# Upfront blood microRNA test in LDCT-reluctant individuals: insights from the biomild trial

**DOI:** 10.1186/s13046-025-03424-5

**Published:** 2025-05-31

**Authors:** Gabriella Sozzi, Federica Sabia, Luigi Rolli, Miriam Segale, Paola Suatoni, Anna Zanghi, Margherita Ruggirello, Alfonso Marchianò, Mattia Boeri, Ugo Pastorino

**Affiliations:** 1 Unit of Epigenomics & Biomarkers of Solid Tumors, Fondazione IRCCS Istituto Nazionale dei Tumori, Via Venezian 1, 20133 Milan, Italy; 2 Unit of Thoracic Surgery, Fondazione IRCCS Istituto Nazionale dei Tumori, Via Venezian 1, 20133 Milan, Italy; 3 Department of Radiology, Fondazione IRCCS Istituto Nazionale dei Tumori, Via Venezian 1, 20133 Milan, Italy

**Keywords:** Lung cancer, MicroRNA, Screening

## Abstract

**Background:**

Low-dose computed tomography (LDCT) lung cancer screening can reduce mortality in high-risk individuals, but many individuals with a heavy smoking history may be reluctant to undergo radiologic examinations. A non-invasive blood test might help overcome this barrier. The BioMILD trial evaluated the combination of a plasma microRNA signature classifier (MSC) and LDCT for personalized lung cancer screening in 4,119 individuals who smoke or used to smoke. Based on BioMILD results, we aim to conduct a projection analysis to estimate the number of early lung cancers that could be detected if MSC were used as an initial screening tool for individuals reluctant to undergo LDCT. This model explores the potential of a biomarker-driven approach to address screening hesitation.

**Main body:**

The analysis focuses on 3,139 volunteers meeting NLST criteria. At baseline, 24.9% tested MSC-positive. Over two years, 63 lung cancer cases were detected, with a significantly higher incidence among MSC-positive participants (4.1% vs. 1.1%, *p* < 0.001). A biomarker-driven approach, where only MSC-positive individuals undergo annual LDCT, was compared to standard LDCT screening for all participants. This strategy could identify 58.7% of lung cancers detected via standard screening, including 56.5% of early-stage cases. Raw cost analysis estimated a per-case lung cancer detection cost of ~€14,000 for the biomarker-driven strategy versus ~€12,000 for standard screening.

**Conclusion:**

Upfront blood MSC test showed a reasonable sensitivity for lung cancer detection, including in early-stage disease, with affordable costs. Such a non-invasive blood test strategy might contribute to improve lung cancer screening endorsement in the high-risk population.

## Background

Long-term results from three randomized trials conducted in the USA (i.e., the National Lung Screening Trial, NLST) and Europe (i.e., the NELSON and the multicenter Italian lung detection, MILD), showed that screening with chest Low-Dose Computed Tomography (LDCT) in individuals with a heavy smoking history, either currently smoking or those who used to smoke, achieve a reduction in lung cancer mortality between 20% and 39%, according to the duration of the intervention [1–3]. While the high detection rate (> 50%) of Stage I-II tumors observed in the LDCT screening arms is certainly a breakthrough, timing of screening intervals, overdiagnosis, management of undetermined lung nodules and false positive results remain open research questions.

Indeed, even though the United States Preventive Services Task Force recently lowered risk criteria of eligibility for screening, the adherence to lung cancer screening in US has only modesty increased [4]. Among the factors that may dissuade eligible individuals to participate in a lung cancer screening program is the concern related to radiation exposure due to prolonged screening rounds and the supplementary exams required in case of suspicious findings [5, 6].

The development of minimally invasive blood biomarkers tests for risk prediction and early detection caught attention from researchers and companies. In fact, a blood based personalized risk approach with a high sensitivity test is expected to have a positive impact as it could avoid even minimal radiation exposure reached by modern ultra-LDCT in lower risk individuals and might contribute to improve lung cancer screening recruitment of heavy smokers aged 55 years or older.

In our previous studies, we developed and validated a microRNA signature classifier (MSC) in plasma samples collected from screening participants. The BioMILD study, involving 4,119 participants, combined LDCT with MSC, demonstrating improved prediction of lung cancer incidence and mortality. For double-negative participants, screening intervals were safely extended to three years without reducing the detection of early-stage, curable cancers [7]. In addition, we recently reported the utility of repeated blood MSC testing in BioMILD participants with suspicious LDCT findings across all screening rounds [8].

Here, we aimed to simulate the benefits, in terms of the number of early-detected lung cancer and the associated costs, that could be achieved by offering an MSC test at baseline to individuals who refuse to undergo the first-line LDCT scan.

## Main text

The BioMILD trial (ClinicalTrials.gov ID: NCT02247453) is a prospective study evaluating the combination of MSC and LDCT to personalize individual risk assessment and screening strategies in 4,119 volunteer individuals who smoke or used to smoke. Details of the clinical trial are reported elsewhere [7, 8]. For present analysis, we selected volunteers eligible for lung cancer screening according to NLST criteria (i.e., aged 55–74 with ≥ 30 pack-years).

The two-year cumulative Kaplan-Meier curves for lung cancer incidence, stratified by baseline MSC categories, were compared using the log-rank test. The percentage of detected lung cancers with specific characteristics, such as stage and histology, was reported overall and stratified by MSC results, with comparisons made using the Chi-Square test. The raw cost of the MSC test was calculated based on reagent prices, equipment depreciation, and personnel costs for analyzing 126 samples per month. The cost of LDCT was estimated based on the reimbursement rate per scan set by the Italian national healthcare system.

The study cohort consisted of 3,139 volunteers, of whom 61% were male and 78% were currently smoking. The median age was 62 years, with a median smoking history of 43 pack-years. At baseline, 781 participants (24.9%) tested positive for the MSC biomarker, while the remaining 2,358 (75.1%) were MSC-negative. Over the two-year period, 63 lung cancer cases were detected. Of these, 46 (73%) were stage I/II, and 42 (66.7%) were adenocarcinomas (Table 1).

**Table 1:** Lung cancer cases detected within 2 years with specific features, stratified by MSC results among biomild volunteers eligible according to the National lung screening trial

		Total	MSC -	MSC+	*p*-value
		3139	2358 (75.1%)	781 (24.9%)	
**LC within 2 years**		63	26 (41.3%)	37 (58.7%)	< 0.0001
**Stage**					
I/II		46 (73.0%)	20 (43.5%)	26 (56.5%)	0.5581
III/IV		17 (27.0%)	6 (35.3%)	11 (64.7%)	
**Histology**					
Adenocarcinoma		42 (66.7%)	21 (50.0%)	21 (50.0%)	0.06
Other		21 (33.3%)	5 (23.8%)	16 (76.2%)	

**Fig. 1:**
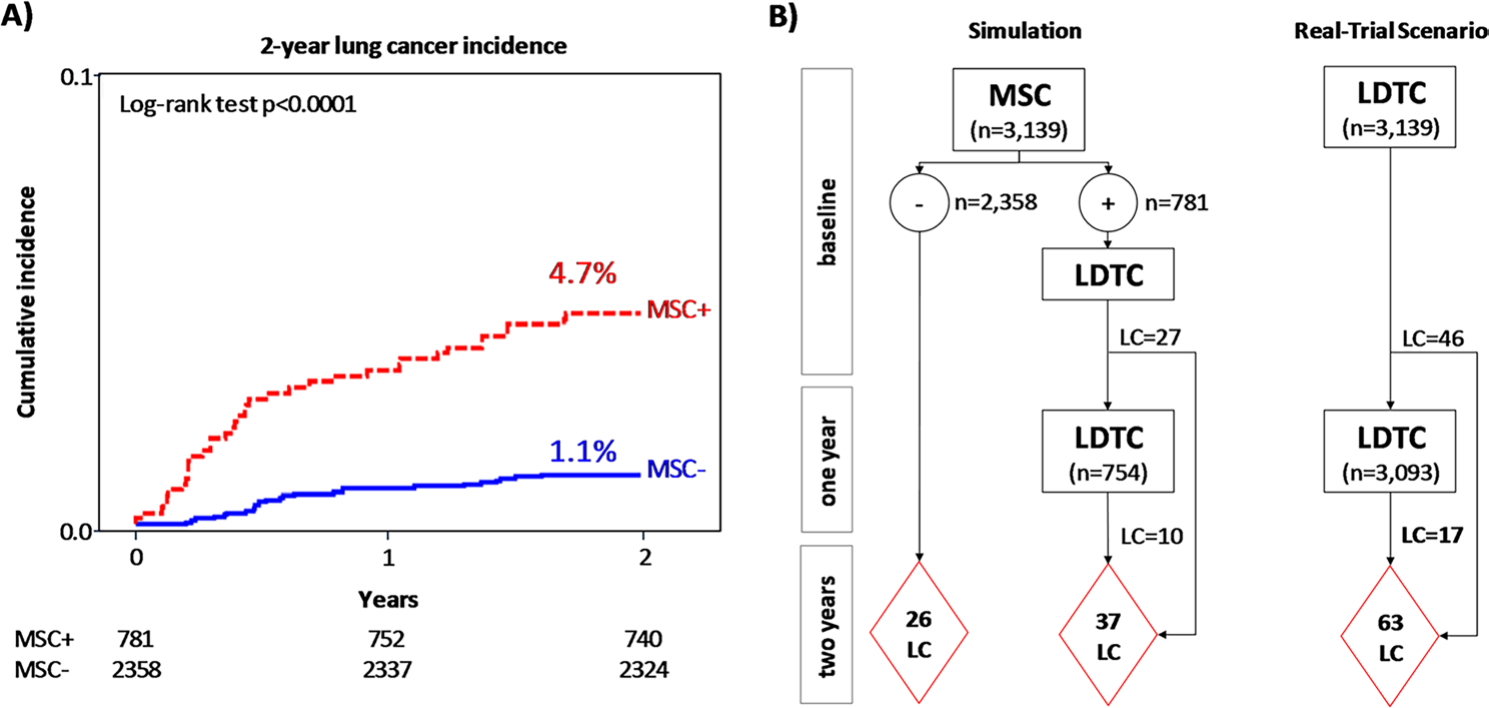
Considering the 3,139 BioMILD volunteers eligible according to the National Lung Screening Trial (i.e., aged 55–74 with pack-years ≥ 30), the 2-year lung cancer incidence curves stratified by MSC test results (**A**), as well as the lung cancer screening paths using up-front MSC (Simulation) or LDCT (Real-Trial Scenario) exams (**B**), are reported

The cumulative lung cancer incidence was significantly higher (*p* < 0.0001) among MSC-positive participants (4.1%) than MSC-negative participants (1.1%) (Fig. 1A). Compared to the standard LDCT-based screening strategy, in which all participants undergo two LDCT screening rounds over two years, in a biomarker-driven lung cancer screening scenario, all 3,139 participants would undergo a baseline blood test, with only the 781 MSC-positive individuals proceeding to annual LDCT screening rounds (Fig. 1B).

All these data suggest that a biomarker-driven approach could identify 58.7% of the lung cancers that would have been diagnosed through standard LDCT screening within two years, including 56.5% of early-stage (I/II) cases. Considering raw costs of €108 per blood test and €120 per LDCT scan, the direct cost per detected lung cancer case would be approximately €14,000 for the biomarker-driven strategy, compared to €12,000 for standard LDCT screening.

## Conclusions

Population-based analyses demonstrate that, although screening rates have increased since 2014, only 5–20% of eligible US adults have completed screening [4]. In addition, 77% of screened individuals did not continue lung cancer screening after a reassuring negative baseline LDCT [9]. Multifactorial barriers to proficient lung cancer screening implementation include volunteers, clinician, and system-factors [5, 6]. In light of this, improvement of self-risk awareness in high risk individuals might increase uptake and adherence to screening.

The present analysis, conducted on screening-eligible participants from the BioMILD cohort, suggests that a blood biomarker-driven lung cancer screening model could offer substantial public health benefits. While the greatest advantages are achieved when all individuals undergo a baseline LDCT, this non-invasive blood test strategy, applied to individuals with a heavy smoking history aged 55 and older who are reluctant to undergo LDCT, maintained reasonable costs and still provided benefits in terms of early cancer detection. However, as our findings are based on projections in people who did agree to LDCT, outcomes may differ in those refusing LDCT and the actual impact of this approach on screening uptake and adherence remains to be confirmed in current clinical practice.

With the advent of NGS platforms and robust analytic methodologies the exploitation of blood-based screening tests has come out as a promising strategy to identify lung cancer high-risk individuals. Genomic profile, epigenomic changes as well as protein and auto-antibody signatures in blood have been developed and are currently under validation in clinical studies.

The seven-autoantibodies EarlyCDT-Lung test, was validated in the Early Diagnosis of Lung Cancer Scotland prospective trial where 12,000 high-risk participants were randomly assigned to the EarlyCDT-Lung testing or standard of care. Only 9.8% of test-positive participants underwent LDCT and a modest reduction (14%) in late-stage tumors was observed at 2-year follow up compared to the standard clinical care group [10]. When assessed in a cohort of participants from the German Lung Cancer Screening Intervention Trial, the test showed 13% sensitivity (4.2% in stage IA) preventing its use in LDCT screening populations [11]. The test is now commercially available (Nodify cdt by Biodesic.inc) for blood-based nodule risk assessment claiming 78% PPV, 98% specificity, 28% sensitivity and has coverage from Medicare in USA.

Circulating tumor DNA-based tests, although promising, have important shortcomings related to the poor limit of detection leading to suboptimal sensitivity for early-stage lung cancer. The Galleri test (GRAIL, Menlo Park, California, USA), a commercial Multi Cancer Early detection (MCED) test evaluating targeted gene methylation, showed an overall sensitivity of 66% in lung cancer patients but only 8.7% for stage I lung cancer in patients referred for cancer investigation of the SIMPLIFY observational cohort study [12].

DELFI, a cell-free fragmentome assay (DELFI diagnostics, Baltimore, USA) has been recently validated in a multicenter, prospective case-control study (DELFI-L101) where enrolled participants generally paralleled those of the population that is eligible for LDCT screening to align with the proposed use of the test in screening trials. A total of 958 lung cancer and non-cancer control participants were evaluated and the test showed 80% overall sensitivity, 71% in Stage I, with 58% specificity [13].

However, performance of both Grail and DELFI tests in true prospective LDCT screening population are still missing. Indeed, the costs of these assays based on high-coverage sequencing of cell-free DNA are remarkable and difficulties to find room for economic support in nation-wide screening programs are foreseen.

In conclusion our low cost, non-invasive, easy-to-implement blood strategy might improve recruitment of high-risk individuals and potentially be offered to those who refuse LDCT screening ultimately reducing barriers to lung cancer screening.

## Data Availability

UP and GS have full access to all of the data in the study and take responsibility for the integrity of the data and the accuracy of the data analysis. All data used in the study are available upon request to the corresponding author.

## Abbreviations

LDCT: low-dose computed tomography
MILD: multicenter Italian lung detection
MSC: microRNA signature classifier
NLST: national lung screening trial
USPSTF: United States preventive services task force
